# Unmasking Essential Thrombocythemia in Heparin-Induced Thrombocytopenia After Coronary Artery Bypass Grafting

**DOI:** 10.1016/j.jaccas.2026.108900

**Published:** 2026-06-17

**Authors:** Sapna Kumari, Basanta Thapa, Sohaib Roomi, Alison Sabados, Grigori Okoev, Prasad Konda, Paul Tolerico, Nishi Patel, Omair Chaudhary, Vishal N. Shah

**Affiliations:** a Department of Internal Medicine, WellSpan York Hospital, York, Pennsylvania, USA; b WellSpan Heart and Vascular Institute, WellSpan York Hospital, York, Pennsylvania, USA; c Department of Pharmacy, WellSpan York Hospital, York, Pennsylvania, USA; d Cancer Care Associates, WellSpan York Hospital, York, Pennsylvania, USA; e Division of Pulmonology and Critical Care, Department of Internal Medicine, WellSpan York Hospital, York, Pennsylvania, USA

**Keywords:** case report, heparin-induced thrombocytopenia, myeloproliferative neoplasm, P2Y_12_ receptor inhibition, thrombosis

## Abstract

**Background:**

Heparin-induced thrombocytopenia (HIT) and *JAK2* V617F–positive essential thrombocythemia (ET) are prothrombotic conditions that complicate postoperative management.

**Case Summary:**

A 68-year-old man underwent coronary artery bypass grafting (CABG) and developed recurrent arterial thrombosis with an abrupt platelet decline prompting evaluation for HIT. Platelet factor 4/heparin immunoassays were positive, whereas serotonin release assays remained negative during potent P2Y_12_ receptor inhibition. Despite discordant laboratory results, a probable diagnosis of HIT was made. In addition to fulfilling other diagnostic criteria, genetic testing revealed a *JAK2* V617F mutation, confirming the diagnosis of ET.

**Discussion:**

Unexplained persistent thrombocytosis in the setting of prior arterial thrombosis should raise suspicion for an underlying myeloproliferative neoplasm. Moreover, the coexistence of *JAK2* V617F–positive ET and probable HIT following CABG confers a heightened thrombotic risk and underscores the diagnostic challenges associated with functional HIT assays in the context of potent P2Y_12_ receptor inhibition.

**Take-Home Message:**

Persistent unexplained thrombocytosis with prior arterial thrombosis should raise suspicion for an occult myeloproliferative neoplasm, whereas postoperative HIT remains a clinical consideration despite negative functional assays, particularly in the context of potent P2Y_12_ receptor inhibition.

## Visual Summary

**Figure:**
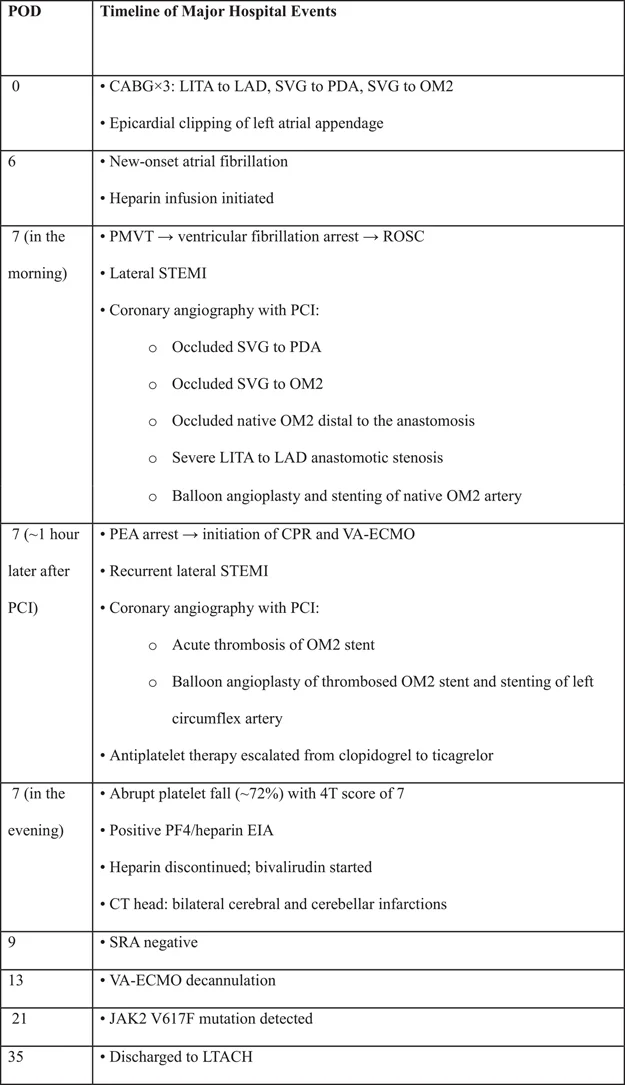

## History of Presentation

A 68-year-old man presented with exertional dyspnea. Coronary angiography demonstrated 80% stenosis of the mid to distal left circumflex artery; 85% and 70% stenosis of the ostial and proximal right coronary artery, respectively; and 75% stenosis of the mid left anterior descending artery (Video 1, Video 2, Video 3). Given multivessel coronary artery disease involving the left anterior descending artery in the setting of diabetes, this low-risk patient—with a Society of Thoracic Surgeons predicted mortality of 1.5%—met a Class I indication for coronary artery bypass grafting (CABG) based on an overall survival benefit.^1^ Following multidisciplinary consensus and shared decision making, he underwent elective triple-vessel CABG with conventional endoscopic saphenous vein harvest and epicardial clipping of the left atrial appendage. The harvested vein grafts were of good quality with sufficient length and caliber. Intraoperatively, the patient required approximately 1.8 times the standard heparin bolus to achieve an adequate activated clotting time. Postoperative echocardiography demonstrated preserved left ventricular function and transient right ventricular dysfunction that improved with a short course of milrinone and bumetanide therapy. Aspirin was initiated on postoperative day (POD) 1, and both clopidogrel and subcutaneous heparin prophylaxis were started on POD 2. On POD 6, new-onset atrial fibrillation prompted initiation of a heparin infusion. On the morning of POD 7, the patient developed polymorphic ventricular tachycardia progressing to ventricular fibrillation arrest in the setting of a lateral ST-segment elevation myocardial infarction, requiring cardiopulmonary resuscitation and defibrillation.

**Take-Home Messages**

• Persistent unexplained thrombocytosis in the setting of prior arterial thrombosis should raise suspicion for an occult myeloproliferative neoplasm.
• Postoperative HIT remains a clinical consideration despite negative functional assays, particularly in the context of potent P2Y_12_ receptor inhibition.

## Past Medical History

The patient had type 2 diabetes mellitus, heart failure with preserved ejection fraction, hypertension, hyperlipidemia, and bilateral renal artery stenosis status post stenting 6 months earlier. He was taking aspirin and clopidogrel; clopidogrel was discontinued 5 days before surgery, whereas aspirin was continued. Despite no history of atrial fibrillation, his CHA_2_DS_2_-VASc score of 4 indicated an elevated stroke risk; therefore, prophylactic left atrial appendage exclusion was performed in anticipation of postoperative atrial fibrillation, consistent with institutional practice.

## Differential Diagnosis

The differential diagnosis included acute coronary ischemia (including graft thrombosis); malignant arrhythmias; mechanical complications or pericardial tamponade; pulmonary embolism; electrolyte or drug-related effects; and underlying hematologic disorders, including hereditary or acquired thrombophilia or myeloproliferative neoplasms.

## Management (Medical/Interventions)

Coronary angiography revealed occlusion of the saphenous vein grafts to the posterior descending and second obtuse marginal (OM2) arteries at their aorto-ostial origins (Videos 4 and 5). Residual flow was present in the native right coronary artery. Approximately 80% stenosis was noted at the left internal thoracic artery–to–left anterior descending anastomosis, with distal TIMI flow grade 2 (Video 6). The proximal and mid segments of the left circumflex artery also exhibited diffuse disease. The native OM2 distal to the graft anastomosis was occluded (Video 7). This culprit segment was treated with predilation using a 2.0-mm noncompliant balloon, followed by a 2.5 × 20 mm drug-coated balloon (Video 8). Owing to residual stenosis >50%, a 2.25 × 22 mm drug-eluting stent was deployed, achieving TIMI flow grade 3. Achieving adequate anticoagulation necessitated high doses of heparin.

Approximately 1 hour later, the patient developed a recurrent lateral ST-segment elevation myocardial infarction, complicated by pulseless electrical activity arrest, requiring cardiopulmonary resuscitation and initiation of veno-arterial extracorporeal membrane oxygenation. Coronary angiography showed acute OM2 stent thrombosis (Video 9). Intravascular ultrasound confirmed a well-expanded stent with no dissection, excluding mechanical causes. The thrombosed stent was treated with serial 2.0-mm noncompliant balloon dilations to restore TIMI flow grade 3. This was followed by placement of 2 overlapping drug-eluting stents in the left circumflex artery (2.5 × 38 mm and 3.5 × 15 mm) to optimize inflow. The final angiographic result is demonstrated in Video 10. Because of concern for clopidogrel nonresponsiveness, antiplatelet therapy was escalated to ticagrelor; aspirin and a heparin infusion were continued.

**Figure 1:**
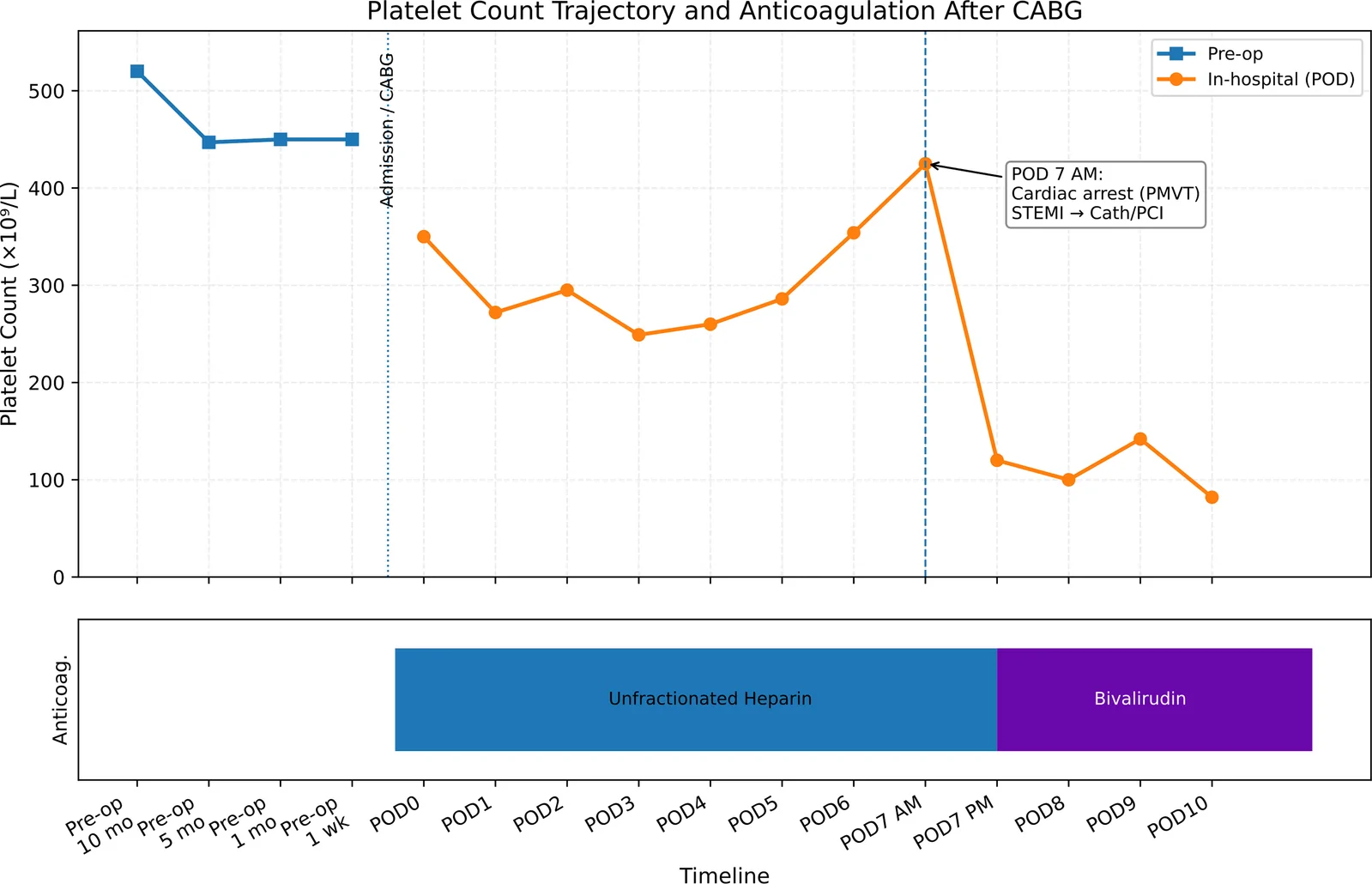
Platelet Count Trend During Hospitalization Platelet counts initially showed preoperative thrombocytosis, followed by an abrupt decline on postoperative day (POD) 7 after exposure to therapeutic heparin during coronary artery bypass grafting (CABG). This approximately 72% decrease was accompanied by new thrombotic events, raising strong clinical suspicion for heparin-induced thrombocytopenia. After discontinuing heparin and initiating bivalirudin, the platelet counts subsequently recovered. Cath = catheterization; PCI = percutaneous coronary intervention; PMVT = polymorphic ventricular tachycardia; STEMI = ST-segment elevation myocardial infarction.

On the evening of POD 7, the platelet count declined abruptly by approximately 72% (from 425 × 10^9^/L to 120 × 10^9^/L) (Figure 1). The 4T score was 7, indicating a high pretest probability of heparin-induced thrombocytopenia (HIT), prompting HIT and prothrombotic laboratory testing (Table 1). The platelet factor 4 (PF4)/heparin enzyme immunoassay (EIA) was positive, with an optical density (OD) of 1.33, and anticoagulation was empirically switched from heparin to bivalirudin. Thereafter, computed tomography of the head demonstrated multifocal bilateral cerebral and cerebellar infarctions; however, the patient had a nonfocal neurologic examination. Although the serotonin release assay (SRA) was negative on POD 9, the high pretest probability and concern for a false-negative functional assay result due to potent P2Y_12_ receptor inhibition with ticagrelor supported a probable diagnosis of HIT. The PF4/heparin EIA OD values were strongly positive on subsequent testing (2.48 on POD 9 and 1.99 on POD 28), despite persistently negative SRAs (Table 2).

**Table 1:** 4T Score Assessment for Heparin-Induced Thrombocytopenia

Category	Criteria	Patient	Points
Thrombocytopenia	>50% fall or nadir ≥20 × 10^9^/L	∼72% fall and nadir >20 × 10^9^/L	2
Timing	Clear onset between 5 and 10 d or platelet fall ≤1 d if prior heparin exposure within 30 d	POD 7 status post CABG	2
Thrombosis	New thrombosis or skin necrosis; acute systemic reaction after heparin bolus	Occluded SVG to OM2 and native OM2 distal to graft anastomosis, acute OM2 stent thrombosis, cerebral and cerebellar infarctions	2
Other causes of thrombocytopenia	Possible	Drug-induced (eg, PPI, clopidogrel, amiodarone, bumetanide), hemodilution, and splenic sequestration post CPB	1
4T score			7

The 4T score was calculated as 7 out of 8, with 2 points assigned for thrombocytopenia, 2 points assigned for timing, 2 points assigned for thrombosis, and 1 point assigned for other potential causes of thrombocytopenia. This total score indicates a high pretest probability of heparin-induced thrombocytopenia.

CABG = coronary artery bypass grafting; CPB = cardiopulmonary bypass; OM2 = second obtuse marginal; PDA = posterior descending artery; POD = postoperative day; PPI = proton pump inhibitor; SVG = saphenous vein graft.

**Table 2:** Serial Laboratory Testing in Suspected Heparin-Induced Thrombocytopenia

POD	Platelet Count (×10^9^/L)	PF4-Heparin EIA OD	SRA Result	P2Y_12_ Receptor Inhibitor	Anticoagulation
6	354	Not ordered	Not ordered	Clopidogrel	Heparin
7 (in the morning)	425	Not ordered	Not ordered	Clopidogrel	Heparin
7 (in the evening)	120	1.33	Negative (48 h later)	Ticagrelor	Bivalirudin
9	83	2.48	Negative (48 h later)	Ticagrelor	Bivalirudin
28	406	1.99	Negative (48 h later)	Ticagrelor	Bivalirudin

Serial laboratory evaluations demonstrated consistently positive PF4/heparin EIAs with strongly positive optical density values, despite persistently negative serotonin release assays during ticagrelor therapy. This discordance between immunologic and functional testing highlights the diagnostic challenge posed by potent P2Y_12_ receptor inhibition, which may lead to false-negative functional assay results.

EIA = enzyme immunoassay; OD = optical density; PF4 = platelet factor 4; POD = postoperative day; SRA = serotonin release assay.

On POD 13, the patient underwent percutaneous veno-arterial extracorporeal membrane oxygenation decannulation. On POD 21, a canonical exon 14 gain-of-function *JAK2* V617F mutation was detected at a variant allele frequency of 10%. In the setting of thrombocytosis, with reactive causes and other myeloproliferative neoplasms excluded, these findings confirmed essential thrombocythemia (ET). Cytoreductive therapy was deferred because platelet counts remained within the target range (<450 × 10^9^/L) and the patient demonstrated clinical improvement. The remainder of the hospital course was complicated by a deep sternal wound infection with dehiscence, necessitating operative debridement, removal of sternal wires, and negative-pressure wound therapy. Echocardiography demonstrated an ejection fraction of 35%.

## Outcome and Follow-Up

On POD 35, the patient was hemodynamically stable, in normal sinus rhythm, with a nonfocal neurologic examination and platelet count of 385 × 10^9^/L and was discharged to a long-term acute care hospital, with subsequent transfer to a rehabilitation facility. Pertinent discharge medications included lifelong low-dose aspirin for ET, ticagrelor for 1 year for coronary stents, apixaban for 3 months for probable HIT, a 6-week course of intravenous antibiotics for deep sternal wound infection, and guideline-directed medical therapy for heart failure indefinitely.

After 2 months, he regained the ability to ambulate with assistance. Neurologic examination remained nonfocal; however, deficits in higher-order cognition and visual fields persisted. Platelet counts were generally <400 × 10^9^/L; therefore, cytoreductive therapy was not initiated. He was discharged home on POD 100 without any further thrombotic events, with instructions for twice-weekly sterile sternal negative-pressure dressing changes.

## Discussion

Specifically, ET is a myeloproliferative neoplasm characterized by excess platelet production and increased thrombotic risk.^2^^,^^3^ In the present case, a history of renal artery stenosis and preoperative thrombocytosis (450-520 × 10^9^/L) may have suggested an underlying prothrombotic state and warranted a high index of suspicion for an occult myeloproliferative neoplasm. The platelet count on the morning of surgery was 350 × 10^9^/L, likely reflecting perioperative hemodilution. Although a bone marrow biopsy was not performed due to elevated procedural risk—particularly hemorrhage—the patient met other diagnostic criteria for ET. Specifically, he demonstrated sustained thrombocytosis (approximately 450 × 10^9^/L for at least 10 years based on outpatient records), absence of an alternative myeloproliferative neoplasm, presence of a *JAK2* V617F mutation (identified in approximately 50% of ET cases), and exclusion of reactive causes of thrombocytosis, including malignancy, iron deficiency, chronic inflammation or infection, and prior splenectomy.^2^^,^^3^ Expert consensus recommends maintaining platelet counts <450 × 10^9^/L in high-risk patients with ET and <600 × 10^9^/L in low-risk patients undergoing major surgery.^3^ According to the revised International Prognostic Score of Thrombosis in ET, this patient was classified as high risk due to age >60 years and the presence of a *JAK2* V617F mutation, corresponding to a per-year thrombosis risk of approximately 2.36% to 4.17%.^3^ The National Comprehensive Cancer Network treatment guidelines recommend low-dose aspirin combined with cytoreductive therapy for high-risk patients, and aspirin alone for patients at low risk.^4^ Although the patient received aspirin, cytoreductive therapy was deferred because postoperative platelet counts remained below the treatment threshold (<450 × 10^9^/L), and interpretation was complicated by probable HIT. Also, the patient had already demonstrated significant clinical improvement by the time the *JAK2* mutation was identified.

Furthermore, HIT is an immune-mediated reaction caused by immunoglobulin G antibodies against PF4-heparin complexes, resulting in platelet activation, thrombocytopenia, and paradoxical thrombosis.^5^ In patients with a 4T score of 4 or higher, immunologic testing followed by functional assays is recommended to confirm or exclude HIT. In our patient, new thrombotic events, along with a >50% platelet drop on POD 7 and a 4T score of 7, prompted evaluation for HIT; however, confirmation was complicated by discordant testing, with positive PF4/heparin EIAs and strongly positive OD values despite persistently negative SRAs during ticagrelor therapy. Because antiplatelet washout was not feasible due to the risk of recurrent stent thrombosis, a probable diagnosis of HIT was made, consistent with reports that potent P2Y_12_ receptor inhibition can cause false-negative functional assays by suppressing adenosine diphosphate–mediated platelet activation.^6^^,^^7^ Additionally, HIT can lower platelets into the normal range, potentially masking an underlying myeloproliferative neoplasm.

The combination of *JAK2* V617F–positive ET and probable HIT after CABG created a highly prothrombotic milieu. Acute thrombosis likely developed in the OM2 graft and the native OM2 distal to the graft anastomosis on the morning of POD 7, coinciding with the patient's initial cardiac arrest. Competitive flow from the native right coronary artery may also have contributed to early posterior descending artery graft failure. Compared with *JAK2*-negative disease, *JAK2*-positive ET, even at low variant allele frequencies, is associated with increased platelet activation, greater PF4 release, higher rates of anti–PF4/heparin antibody formation, and an elevated incidence of HIT (approximately 17% vs 4%). These findings support a potential mechanistic link between *JAK2*-driven myeloproliferation and susceptibility to HIT and related thrombosis.^8^^,^^9^ Heparin resistance has also been reported in *JAK2* V617F–positive ET, likely due to PF4-mediated heparin neutralization and increased thrombin generation.^10^

## Conclusions

This case illustrates that occult *JAK2* V617F–positive ET in a patient with persistent unexplained thrombocytosis may increase thrombotic risk with heparin exposure, reflecting overlapping *JAK2*-driven prothrombotic biology and HIT susceptibility. It also highlights the diagnostic challenge of confirming HIT during potent P2Y_12_ receptor inhibition, where false-negative functional assays require management guided by high clinical suspicion.

## Funding Support and Author Disclosures

The authors have reported that they have no relationships relevant to the contents of this paper to disclose.

## Appendix

Video 1 Preoperative Coronary Angiography Showed 80% Stenosis of Mid Left Circumflex Artery

Video 2 Preoperative Coronary Angiography Showed 85% and 70% Stenosis of Ostial and Proximal Right Coronary Artery, Respectively

Video 3 Preoperative Coronary Angiography Showed 75% Stenosis of Mid Left Anterior Descending Artery

Video 4 Occlusion of Saphenous Vein Graft Supplying Posterior Descending Artery

Video 5 Occlusion of Saphenous Vein Graft Supplying Second Obtuse Marginal Artery

Video 6 Severe Stenosis at Left Internal Thoracic Artery–to–Left Anterior Descending Artery Anastomosis

Video 7 Distal Occlusion of Saphenous Vein Graft Supplying Second Obtuse Marginal Artery and Native Second Obtuse Marginal Artery Distal to Coronary Graft Anastomosis

Video 8 Percutaneous Coronary Intervention of Native Second Obtuse Marginal Artery Including Balloon Angioplasty

Video 9 Acute Stent Thrombosis of Second Obtuse Marginal Artery

Video 10 TIMI Flow Grade 3 Restored Within Stent
